# A Warrior Society: Data From 30 Countries Show That Belief in a Zero-Sum Game Is Related to Military Expenditure and Low Civil Liberties

**DOI:** 10.3389/fpsyg.2018.02645

**Published:** 2019-01-09

**Authors:** Joanna Różycka-Tran, Paweł Jurek, Michał Olech, Jarosław Piotrowski, Magdalena Żemojtel-Piotrowska

**Affiliations:** ^1^Institute of Psychology, University of Gdańsk, Gdańsk, Poland; ^2^Department of Psychology, Cardinal Stefan Wyszyński University in Warsaw, Warsaw, Poland

**Keywords:** Belief in a Zero-Sum Game Scale, military expenditure, democracy, civil liberties, multilevel modeling (MLM)

## Abstract

The aim of this paper was to investigate the relationship between a perceived antagonistic view of social relations (as a struggle for limited resources), measured by the Belief in a Zero-Sum Game (BZSG) Scale, national military expenditure, and civil liberties. We used multi-level modeling to analyze data on 5,520 participants from 30 countries, testing the hypothesis that a country’s level of militarization and civil liberties would be associated with its people’s belief in a zero-sum game. We hypothesized that BZSG is more typical of countries that try to gain more resources or defend their interests and thus have high military expenditure but low civil liberties. The results confirmed the stated hypothesis and showed that a country’s high military expenditure and low level of civil liberties correlates positively with citizens’ BZSG. The use of multi-level modeling to account for within- and across-country variation is a main contribution of the study. In conclusion, the reported triad of individual beliefs, military expenditure, and civil liberties seems to be beneficial in linking individual-level data with national-level indices that have major importance for the wellbeing of the world.

## Introduction

We can observe many wars and military interventions ongoing throughout the world in recent years. The global list of violent conflicts is long, including around 50 conflicts involving more than 60 countries and at least 370 guerrilla groups (Peace Research Institute Oslo [PRIO], 2017; Uppsala Conflict Data Program [UCDP], 2017), and in 2017 alone these conflicts resulted in the deaths of more than 200,000 people. The Fragile States Index (Fund for Peace, 2017) is a scale of the military threat that a country faces and indicates that almost 125 countries are at risk of war.

Although policymakers influence public attitudes, military intervention can only be carried out when there is support for, or acceptance of war at least in democracies (Foyle, 2004). Many studies have found that the specific beliefs in a given culture are connected with government policy – e.g., societal cynicism, which represents a generalized belief that the social system and institutions of a society are hostile toward its members (Bond et al., 2004; Stavrova and Ehlebracht, 2015). Such findings indicate that attitudes and beliefs can affect people’s willingness to engage in war-related behaviors. On the other hand, persistently high military spending may also contribute to such willingness. Hence the links between political/economic systems and psychological processes are an important target for investigation in relation to global militarization.

There are a number of complex interrelated individual- and country-level factors conducive to war, and they can be approached from various disciplinary perspectives. At the country level, the most important factors are the political system and the economic situation reflected in, for example, expenditure on militarization. Also, the relationship between military spending and human rights is one of the most prominent issues in political economics. There is some evidence that an increase in military spending significantly reduces human rights (e.g., Vadlamannati and Pathmalal, 2008). With respect to institutional factors, the literature shows that level of democracy is a key determinant of human rights (Davenport and Armstrong, 2004), especially civil liberties, as subcomponents of the democracy index. Thus, from the perspective of a country’s readiness for war, it is worthwhile investigating the level of democracy (i.e., civil liberties), together with military expenditure.

However, from a psychological perspective, Bar-Tal (2000) claims that conflicts between nations or societies erupt due to perception, or when goals or actions are perceived as mutually incompatible. Kelman (1997, p. 219) defined international conflict as an “intersocietal process driven by collective needs and fears, rather than entirely the product of rational calculation of objective national interests.” Experimental studies have confirmed that people making judgments about whether to oppose or to support war use the logic of deontology rather than the logic of instrumental rationality (Ginges and Atran, 2011). This means that militaristic conflicts are sometimes shaped by emotions and subjective beliefs rather than by rational objective circumstances and interests. Such findings are really alarming, suggesting that choices about deadly intergroup violence are not based on fully rational calculations, but may reflect deontological reasoning, leading to judgments that are insensitive to risks and outcomes.

The psychological variables that have been identified as important drivers of war (Cohrs and Moschner, 2002) include personal values (Mayton et al., 1999; Bègue and Apostolidis, 2000) and generalized ideological attitudes (Pratto et al., 1994; Nelson and Milburn, 1999; McFarland, 2005). Cohrs et al. (2005) analyzed psychological determinants of generalized militaristic attitudes and attitudes to specific wars, namely the Kosovo war in former Yugoslavia, the Iraq war against Saddam Hussein, and the Afghanistan war against global terrorists and the Taliban regime. Equivalent path analyses showed that the effect of conservation values on attitude to the Afghanistan war, Kosovo war, and generalized militaristic attitudes was mediated by right-wing authoritarianism (RWA), whereas the effects of self-enhancement values were mediated by social dominance orientation (SDO). In all models, RWA and SDO were predictors of militaristic attitudes (Cohrs et al., 2005). Bizumic et al. (2013) confirmed the finding of Cohrs et al. (2005) that RWA and SDO predicted both attitude to war and peace and intention to engage in warlike activities. People appear willing to engage in pro-war behaviors, such as fighting for one’s country (in defense of its interests, including purely economic interests), when they value war.

McFarland (2005) reported similar findings in relation to the effects of RWA and SDO on support for attacking Iraq: RWA strengthened support for the attack by intensifying the perception that Iraq threatened America, whereas SDO increased support by reducing concern for the likely human cost of the war (the loss of innocent lives). Authoritarianism intensifies the perception of external threat (and activates the fear of in-group disunity), whereas social dominance strengthens concern for identification with a powerful in-group.

Another psychological factor connected with war is the specific beliefs shared by individuals (Herrmann et al., 1999; Eidelson and Eidelson, 2003; Vollhardt, 2009). Such collective beliefs, shared worldviews, or socially shared cognitions (see Thompson and Fine, 1999) can play a destructive role in intergroup relations (Kelman, 1987; Brewer and Miller, 1996), and the zero-sum aspects of competition over scarce resources can lead to intractable conflict between contending groups (Bar-Tal, 2000). According to the group conflict theory (Sherif, 1966), no peaceful solution is possible from a zero-sum viewpoint (the outcome will instead reflect a balance of power), so competing groups refuse to make concessions to each other and support investment of societal resources in the strengthening of militaristic institutions (Hagai et al., 2013). Support for resolving conflicts through military interventions may be greater amongst communities sharing such beliefs.

In this paper, we investigate belief in a zero-sum game and its possible associations with a country’s militarization and civil liberties as subcomponents of the democracy index. Belief in a zero-sum game is a “general belief about the antagonistic nature of social relations, shared by people in a society or culture and based on the implicit assumption that a finite amount of goods exists in the world, in which one person’s winning makes others the losers, and vice versa—a relatively permanent conviction that social relations are like a zero-sum game” (Różycka-Tran et al., 2015, p. 526), and can be measured using the Belief in a Zero-Sum Game (BZSG) scale. The BZSG scale can be used for cross-cultural comparisons, as its cross-cultural measurement invariance was confirmed by analysis of data from 36 countries (Różycka-Tran et al., 2017).

The results also revealed an isomorphic factor structure of the BZSG scale, defined in terms of the equivalence factor structure at both the individual and country levels (Różycka-Tran et al., 2018a).

Previous studies have shown that general belief in the antagonistic nature of social relations tends to be higher in countries with a lower standard of living, where citizens have to compete for resources—that is, the BZSG scale is negatively related to the human development index (HDI) and income, but positively to the collectivism where people’s interests are more interdependent (Różycka-Tran et al., 2015). Our results are compatible with other studies showing that income per capita is systematically and negatively correlated with civil war (Fearon and Laitin, 2003). A review of the literature on social conflict and economic development confirms that higher standards of living reduce the probability of conflict—that is, countries with lower incomes are more willing to join militaristic conflicts or display a preference for dealing aggressively with out-group threats in order to gain more resources or defend their interests (see Ray and Esteban, 2017).

However, such findings do not take into account countries with high GDP (e.g., the United States), which also spend more on militarization. In fact, military spending is related to several factors, such as political, economic, ecological, and cultural conditions. For instance, number of conflicts is related to the interaction between climate stress (extreme temperature) and affluence (Van Lange et al., 2017; Van De Vliert and Conway, 2018): in cultures with high climatic stress, affluence is associated positively with adaptation to the environment, resulting in higher creativity. On the other hand, higher ecological stress combined with low affluence is related to a culture of conflict (Van De Vliert and Conway, 2018). In addition, higher spending on militarization is related to the masculinity dimension—i.e., prioritizing values typical for male roles, like achievement, financial success, or heroism (Hofstede et al., 2010). Among countries from the top of the Global Militarization Index (GMI), there is an overrepresentation of countries with a relatively low democracy level. There is also a negative relationship between hunger and militarization (Mutschler, 2016) and a negative relationship between oil prices and militarization (Mutschler, 2017). Even though militarization is higher among rather unstable and undemocratic societies: it requires financial resources, especially in the case of heavy weapons spending.

Taking into account different correlates of militarization, we decided to focus on investigating the relationship between country-level indices of real military expenditure and civil liberties as subcomponents of the democracy index (with income and HDI as controlled variables), and on individual-level belief in the antagonistic nature of social relations in 30 countries. We hypothesized that average BZSG measured at the individual level would be positively related to militarization indexed by objective military expenditure at the country level (hypothesis 1), and negatively related to civil liberties (hypothesis 2). Furthermore, we hypothesized that militarization and civil liberties are respectively positively and negatively associated with BZSG using a multi-level modeling approach (hypothesis 3).

## Materials and Methods

### Participants and Procedure

Data were collected from 5,520 college students (40% male) in 30 countries.^1^ The mean age of students was 21.03 years (*SD* = 2.34). All samples were composed of college students studying social sciences or business management, recruited as volunteers; some received a course credit for participation. Participants filled out a pencil and paper version of the BZSG scale and were asked also to report their age and gender. Participants were assured that their data would remain anonymous and confidential. Participants’ consent was obtained by virtue of survey completion. The study has been conducted according to the principles expressed in the Declaration of Helsinki. All procedures were approved by each participating university’s ethics committee.

To ensure comparability of the samples in terms of age and educational level, all data from respondents under 18 years or over 30 years of age were removed from the collection. As a result, the average age of national sub-samples ranged from 19.13 years (Japan) to 22.67 years (Latvia) (for details, see Table 1).

**Table 1:** Samples composition, Cronbach’s alphas and descriptive statistics of the BZSG scores in 30 countries.

Country	*N*	% of men	Age		BZSG raw score			BZSG standardized factor score	
					
			*M*	*SD*	*Alpha*	*M*	*SD*	*M*	*SD*
Belgium	181	30	19.56	1.82	0.84	2.98	0.87	–0.25	0.77
Brazil	201	39	20.52	3.21	0.78	2.83	0.83	–0.36	0.73
Bulgaria	164	40	21.55	2.40	0.86	2.98	0.97	–0.22	0.83
Colombia	103	46	19.31	1.70	0.91	2.96	1.11	–0.68	0.61
Czech Republic	179	32	21.39	2.60	0.87	2.55	0.75	–0.23	0.77
Estonia	275	34	21.56	3.15	0.89	3.01	0.92	0.23	0.78
Hungary	205	31	21.01	1.69	0.86	2.80	0.91	–0.35	0.79
India	191	33	22.42	1.10	0.80	3.75	1.05	0.38	0.95
Indonesia	200	50	21.38	1.65	0.88	3.11	1.05	–0.08	0.91
Iran	201	50	21.28	1.53	0.79	3.81	1.11	0.40	0.96
Japan	166	50	19.13	1.15	0.83	3.21	0.91	–0.02	0.79
Kazakhstan	209	32	20.24	1.76	0.87	3.31	0.90	0.03	0.77
Kenya	130	40	21.66	1.98	0.87	3.04	1.10	–0.14	0.92
Korea Republic	192	46	21.90	1.75	0.89	3.47	0.86	0.03	0.83
Latvia	125	40	22.67	2.50	0.95	3.96	1.09	0.14	0.74
Malaysia	200	50	21.96	1.22	0.89	3.63	1.04	0.47	0.88
Nepal	197	50	22.27	3.12	0.76	3.79	0.86	0.28	0.86
Pakistan	195	50	21.46	1.59	0.86	3.48	0.99	0.43	0.76
Poland	222	40	21.93	2.60	0.91	2.87	1.04	0.15	0.82
Portugal	173	30	20.93	2.80	0.85	2.87	0.89	–0.30	0.90
Romania	195	49	20.72	1.74	0.90	3.32	1.08	–0.30	0.76
Russia	172	36	20.80	2.19	0.88	3.05	0.82	0.03	0.94
Serbia	188	43	21.01	2.84	0.88	3.48	1.10	–0.12	0.70
Slovak Republic	189	32	20.99	1.10	0.83	3.00	0.73	0.13	0.95
South Africa	180	34	20.20	1.71	0.83	3.31	0.95	–0.25	0.64
Spain	188	50	20.64	2.74	0.88	3.45	1.10	0.20	0.93
Ukraine	142	29	20.19	1.84	0.88	3.11	0.94	–0.11	0.81
United Kingdom	208	32	19.61	1.65	0.86	2.96	0.83	–0.23	0.71
United States	106	38	21.47	2.57	0.88	3.33	0.89	0.07	0.75
Vietnam	243	49	20.60	2.40	0.84	3.63	0.96	0.30	0.82
**Total**	**5,520**	**40**	**21.03**	**2.34**	**0.87**	**3.24**	**1.02**	**0**.**01**	**0.86**


In most national sub-samples, removal of young and mature students did not result in the loss of more than 5 percent of data; the exceptions were Latvia (45%), Colombia (26%), Kenya (20%), and Estonia (10%). Also, as can be seen in Table 1, the proportion of men in the sub-samples varied from 29% (Ukraine) to 50% (Indonesia, Iran, Japan, Malaysia, Nepal, Pakistan, and Spain), which strains the assumption that sub-samples were nationally representative. To some extent, the gender distribution of sub-sample reflects the proportion of men and women studying business management or social sciences at universities in the relevant country. In any event, due to national differences in age and gender distribution, both variables were included as covariates in the tested models.

### Measures

#### Belief in a Zero-Sum Game

The BZSG scale (Różycka-Tran et al., 2017) consists of eight items (see Table 2) reflecting beliefs about the antagonistic nature of competition over scarce resources^2^. The BZSG scale was translated into 20 languages (Armenian, Bulgarian, Chinese, Czech, Estonian, Flemish, French, Georgian, German, Hungarian, Japanese, Polish, Romanian, Serbian, Slovakian, Portuguese, Spanish, Russian, Ukrainian, and Vietnamese).

**Table 2:** Results of confirmatory factor analysis: standardized factor loadings, intercepts and variances.

Item	Factor loading	Intercept	Variance
(1) Successes of some people are usually failures of others.	0.64	2.29	0.60
(2) If someone gets richer it means that someone else gets poorer.	0.78	2.20	0.39
(3) Life is so devised that when somebody gains, others have to lose.	0.81	2.26	0.34
(4) In most situations interests of different people are inconsistent.	0.50	2.98	0.75
(5) Life is like a tennis game - a person wins only when others lose.	0.75	2.15	0.43
(6) When some people are getting poorer it means that other people are getting richer.	0.79	2.45	0.38
(7) When someone does much for others he or she loses.	0.54	1.94	0.71
(8) The wealth of a few is acquired at the expense of many.	0.61	2.49	0.72


*N = 5,520.*

Bilingual individuals working in psychology as academics at universities used the back translation procedure to create national versions of the scale. The English version of the BZSG was used as the basis for all translations. Responses were given using a six-point Likert scale (1 = strongly disagree to 6 = strongly agree). After demonstrating the cross-country measurement invariance of the BZSG (see Results section) and computing standardized individual BZSG scores, we carried out confirmatory factor analysis (CFA) of data from the whole sample. Aggregated standardized factor scores were used to compute country-level BZSG.

#### Militarization of the Country

The militarization of a country can be indexed using military expenditure as a percentage of government spending. This indicator is independent of GDP, which influences absolute military expenditure (Pereira, 2004), and is used to provide objective global rankings of militarization. We used 2015 data on military expenditure as a percentage of central government expenditure. These data were available for 26 of the 30 countries (World Bank, 2017). A more complex measure of a country’s militarization is the GMI, which relates a state’s military facilities to various other indicators. It has three components: (a) expenditure, or the comparison of a country’s military expenditure with its GDP and its health expenditure; (b) personnel, or the number of military personnel relative to the number of physicians and the overall population; and (c) heavy weapons, or the number of heavy weapons available per head of population (for details, see Grebe and Mutschler, 2015). We used 2015 data for all 30 countries (Bonn International Center for Conversion [BICC], 2017).

#### Civil Liberties as Subcomponents of the Democracy Index

Civil liberties are closely related to the principle of the protection of human rights, these including freedom of speech, freedom of expression, a free press, freedom of religion, the rights to assembly and association, and the right to due judicial process. Civil liberties along with the electoral process and pluralism, the functioning of government, and political participation and political culture - are the basic components of the Democracy Index, which is an index compiled by the Economist Intelligence Unit (EIU). We used 2015 data for all 30 countries (EIU, 2016), including all components of the Democracy Index, paying special attention to civil liberties.

#### Control Variables

Because previous research has shown that gender and age are related to BZSG at the individual level, and it has also been reported that per capita GDP, HDI, and BZSG are related at the country level (see Różycka-Tran et al., 2015), we decided to control these variables. Per capita GDP is a country’s GDP divided by its population and is an indicator of a country’s standard of living. HDI is a composite statistic of lifespan, educational level, and per capita GDP, used to classify countries into four tiers of human development.

### Analytical Strategy

We needed firstly to determine whether the BZSG scale was used in a comparable manner and represented the same construct in the 30 countries we investigated—that is, whether it demonstrated measurement invariance. We assessed the scale’s cross-cultural equivalence through multigroup confirmatory factor analysis (MGCFA). The factorial structure of the scale was assessed separately for each country using CFA. We assessed models’ goodness of fit using commonly used criteria: CFI > 0.90 and RMSEA < 0.08 (e.g., Brown, 2015). However, Kenny et al. (2015) showed that RMSEA often underestimates fit when the number of degrees of freedom is small, especially when this relates to small sample size, and so we decided to use a more liberal RMSEA criterion (RMSEA < 0.10), which is also in line with MacCallum et al. (1996), who suggest that values of RMSEA between.08 and.10 indicate mediocre fit. Because we had item-level, ordered categorical data, we used the diagonally weighted least squares estimator with robust standard errors and a mean-adjusted test statistic (WLSM) to estimate CFA parameters (DiStefano and Morgan, 2014). Next, we tested the measurement invariance of the eight-item version of the BZSG scale across 30 countries.

Multigroup confirmatory factor analysis usually requires estimates of three types of invariance, which are defined by the parameters that are constrained to be equal across samples (e.g., Milfont and Fisher, 2010). Configural invariance requires that a given set of indicators is underlain by the same latent variables with the same pattern of factor loadings; metric (weak) invariance requires that factor loadings are equal across the groups; and scalar (strong) invariance requires that factor loadings and all intercepts are equal across the groups (see Milfont and Fisher, 2010; Beaujean, 2014). It is also possible to assess partial invariance, which is sufficient to allow for group comparisons. Partial invariance is established when the parameters of at least two indicators per construct are equal across groups (Byrne et al., 1989). Analyses were computed with lavaan, the R package for structural equation modeling (Rosseel, 2012). We started investigating measurement invariance by testing for configural invariance across national sub-samples, using the same criteria as in the case of CFAs for each group separately. To identify metric and scalar measurement invariance, we used the cut-off criteria suggested by Rutkowski and Svetina (2014): ΔCFI ∼0.02 and ΔRMSEA ∼0.03.

We computed bivariate correlations among the study variables to explore relations between variables at the country level and to test the hypothesis about the relationships between selected country-level variables (i.e., militarization expenditure and civil liberties), with the individual-level belief in a zero-sum game. We carried out multilevel modeling (MLM; e.g., Hox, 2010) with data from 5,520 individuals (Level 1) across 30 countries (Level 2). In all analyses, standardized factor scores for the BZSG, as well as log transforms of per capita GDP, Democracy Index, electoral process, and pluralism and civil liberties, were used. Analyses were carried out with nlme, the R package for fitting multilevel models (Finch et al., 2014).

The multilevel analyses were specified sequentially by incorporating additional predictors into each successive model to produce nested models that could be compared statistically. Models were fitted using maximum likelihood (ML) estimation. The fit of nested models was assessed using -2 log likelihood (-2LL) and Akaike’s information criteria (AIC), where lower values indicate better fit (Finch et al., 2014). We determined whether a model represented an improvement over the prior model using Δχ^2^, computed from the difference in the -2LL of two nested models.

Country served as the grouping variable in all models. Model 1 was specified as a baseline model with no independent variable. This model provided estimates of the residual and intercept variance when only the clustering by country was under consideration. The baseline model allowed us to determine whether mean BZSG scores differed across the 30 countries. It also provided the intraclass correlations (ICCs), which relate within-country similarity in BZSG scores to the total variation in individual beliefs across all countries. A high ICC value signifies that the scores of individuals are not statistically independent within countries, and that the nested design should therefore be taken into account and a multilevel model calculated.

Models 2, 3a, 3b, and 4 involved random coefficients and fixed predictors. Model 2 built on the previous model by including participants’ gender and age (fixed-effect predictors at the individual level). Models 3a and 3b each incorporated one fixed-effect predictor at the country level (military expenditure and civil liberties respectively). Model 4 incorporated both fixed-effect predictors at the country level. Thus, in Models 2, 3a, 3b, and 4, the impact of gender on the BZSG is allowed to vary from one country to another. All models’ fit statistics were compared to determine whether the addition of each subsequent predictor or block of predictors enhanced model fit.

We tested all the above-mentioned models with and without control variables at the country level (per capita GDP and HDI). However, as Becker et al. (2016) have cautioned that inclusion of control variables may hamper the analyses by unnecessarily consuming degrees of freedom and potentially biasing findings, and given that the control variables were not associated with the dependent variable, we report country-level results from analyses without control variables.

## Results

### Measurement Invariance of the BZSG

First we tested a one-factor BZSG structure based on the pooled covariance matrix. Factor loadings, intercepts, and error variances for each item in the pooled international sample (*N* = 5,520) are presented in Table 2. The model was a good fit to data: χ^2^ = 465.48, *df* = 20, CFI = 0.98, TLI = 0.98, RMSEA = 0.064 (90% CI: 0.055–0.072). The BZSG factor scores had very good reliability (McDonald’s ω = 0.87).

Next we calculated descriptive statistics (mean, standard deviation, and Cronbach’s alpha) for the BZSG separately for each country. As can be seen in Table 1, the Cronbach’s alpha values indicate that the BZSG was reliable in all national sub-samples. We also conducted separate CFAs testing a one-factor model of the BZSG. As can be seen in Table 3, the CFI values suggested good fit in all 30 countries, whereas the RMSEA values were less clearly supportive. A large number of RMSEA confidence intervals exceeded the liberal cut-off point of 0.10. In evaluating these results, it is important to take into account the low complexity of the tested model. The BZSG scale measures one factor and consists of a relatively small number of items. Kenny et al. (2015) state that RMSEA, which is amongst the most commonly used statistics for evaluating model fit, should not be used with small *df* models and, on this basis, we decided to use CFI to evaluate national model fit.

**Table 3:** Global fit measures for the single sample CFAs (df = 20) of BZSG scale in 30 countries.

Country	*N*	χ^2^	*p*	CFI	RMSEA	RMSEA lower 90% CI	RMSEA upper 90% CI
Belgium	181	51.17	0.00	0.90	0.093	0.062	0.125
Brazil	201	39.83	0.01	0.93	0.070	0.038	0.102
Bulgaria	164	37.25	0.01	0.94	0.073	0.034	0.109
Colombia	103	27.77	0.11	0.96	0.062	0.000	0.112
Czechia	179	50.55	0.00	0.92	0.093	0.061	0.125
Estonia	275	33.62	0.03	0.98	0.050	0.016	0.078
Hungary	205	37.62	0.01	0.95	0.066	0.032	0.098
India	191	36.08	0.02	0.96	0.065	0.028	0.099
Indonesia	200	30.21	0.07	0.98	0.051	0.000	0.086
Iran	201	53.74	0.00	0.91	0.092	0.063	0.122
Japan	166	35.91	0.02	0.93	0.069	0.030	0.105
Kazakhstan	209	33.61	0.03	0.97	0.057	0.018	0.090
Kenya	130	30.47	0.06	0.97	0.064	0.000	0.107
Korea Republic	192	27.45	0.12	0.98	0.044	0.000	0.081
Latvia	125	31.34	0.05	0.98	0.068	0.000	0.111
Malaysia	200	43.48	0.00	0.95	0.077	0.045	0.108
Nepal	197	46.59	0.00	0.89	0.082	0.052	0.113
Pakistan	195	54.03	0.00	0.93	0.094	0.064	0.124
Poland	222	37.18	0.01	0.97	0.062	0.029	0.093
Portugal	173	34.45	0.02	0.95	0.065	0.024	0.101
Romania	195	46.91	0.00	0.94	0.083	0.052	0.114
Russia	172	34.45	0.02	0.94	0.065	0.024	0.101
Serbia	188	26.97	0.14	0.99	0.043	0.000	0.081
Slovak Republic	189	30.32	0.06	0.96	0.052	0.000	0.088
South Africa	180	38.70	0.01	0.95	0.072	0.037	0.106
Spain	188	46.73	0.00	0.94	0.085	0.053	0.116
Ukraine	142	24.47	0.22	0.98	0.040	0.000	0.087
United Kingdom	208	42.13	0.00	0.94	0.073	0.042	0.104
United States	106	21.72	0.36	0.99	0.029	0.000	0.091
Vietnam	243	28.93	0.09	0.98	0.043	0.000	0.075


Finally, we conducted a three-level measurement equivalence test. Table 4 presents the global fit coefficients for the three levels of measurement invariance: configural, metric, and scalar. First, we established that the eight-item version of the scale displayed configural invariance and metric invariance across all the countries, according to the cut-off criteria suggested by Rutkowski and Svetina (2014). These results allow us to conclude that the BZSG scale has metric invariance across all 30 countries. In view of the lack of full scalar invariance, we tested for partial scalar invariance, releasing three items (1st, 4th, and 7th) that varied most between countries. Results support the conclusion about the partial scalar invariance of BZSG scale across all 30 countries.

**Table 4:** Global fit measures in measurement invariance tests for eight-items version of the BZSG scale.

Level of invariance	χ^2^	*df*	CFI	RMSEA	Δ CFI	Δ RMSEA
Configural invariance (equal form)	1347.47	600	0.971	0.083	–	–
Metric (weak) invariance (equal factor loadings)	1982.86	803	0.954	0.090	0.017	0.02
Partial scalar (strong) invariance (equal indicator intercepts)^∗^	2669.91	919	0.932	0.102	0.022	0.012
Scalar (strong) invariance (equal indicator intercepts)	3993.62	1006	0.885	0.127	0.069	0.037


*30 countries; ^∗^Intercepts for item 1, 4, and 7 were released.*

### Descriptive Statistics and Correlations Between Variables at the County Level

Descriptive statistics and bivariate correlations between variables at the country level are presented in Table 5.

**Table 5:** Descriptive statistics and correlations between variables at the country level.

Variable	Min.	Max.	*M*	*SD*	1	2	3	3a	3b	3c	4	5	6	6a	6b	6c	6d
(1) BZSG	–0.67	0.47	–0.01	0.28													
(2) Military gov. expenditures ^A^	2.12	15.89	6.88	4.12	0.37^†^												
(3) Global Militarisation Index	455.33	798.48	618.61	87.60	0.09	0.54**											
(3a) Military expenditures	4.68	5.84	5.16	0.31	0.39*	0.86**	0.69**										
(3b) Military personnel	3.06	6.03	4.44	0.75	–0.01	0.38^†^	0.90**	0.47**									
(3c) Heavy weapons	1.47	3.24	2.32	0.43	–0.03	0.26	0.73**	0.39*	0.44*								
(4) GDP per capita ^B^	743	56207	14173.46	13951.76	–0.24	–0.28	–0.04	–0.38*	0.01	0.41*							
(5) Human Development Index	0.55	0.92	0.78	0.11	–0.21	–0.23	0.19	–0.30	0.16	0.54**	0.49**						
(6) Democracy Index ^B^	2.16	8.31	6.53	1.72	–0.28	–0.31	–0.27	–0.46*	–0.15	–0.10	0.67**	0.59**					
(6a) Electoral process and pluralism ^B^	0.00	9.58	7.49	3.12	–0.38*	–0.43*	–0.24	–0.44*	–0.14	–0.08	0.59**	0.54**	0.88**				
(6b) Functioning of government	2.14	y8.21	6.15	1.71	–0.21	–0.30	–0.33^†^	–0.43*	–0.20	–0.25	0.48**	0.34^†^	0.85**	0.76**			
(6c) Political participation	2.78	8.33	5.83	1.34	–0.20	–0.07	–0.19	–0.28	–0.13	–0.08	0.32^†^	0.29	0.70**	0.50**	0.51**		
(6d) Political culture	2.50	8.75	5.75	1.63	–0.07	–0.18	–0.19	–0.48*	–0.01	–0.12	0.53**	0.50**	0.73**	0.45*	0.54**	0.47**	
(6e) Civil liberties ^B^	1.47	9.41	7.41	2.23	–0.39*	–0.42*	–0.29	–0.46*	–0.18	–0.13	0.60**	0.55**	0.90**	0.95**	0.71**	0.54**	0.52**


*BZSG, An average Belief in a Zero-Sum Game factor score in a given country; Military gov. expenditures, % of central government expenditures on militarization in 2015, GDP per capita, Gross Domestic Product per capita (current US$) in 2015; ^*A*^*N* = 26; ^*B*^For the purpose of correlation analysis a log transform for this indexes was used; ^∗^*p* < 0.05; ^∗∗^*p* < 0.01; ^†^*p* < 0.10.*

As can be seen, military expenditure was positively correlated with average BZSG score, while electoral process and pluralism and civil liberties were negatively correlated. However, the variance inflation factor (VIF) values for electoral process and pluralism and civil liberties were 4.55, indicating that use of both predictors in one analysis is inappropriate due to collinearity. As a consequence, we dropped electoral process and pluralism from further analyses and kept civil liberties due to its higher correlation with BZSG. Furthermore, as shown in Table 5, variables such as the GMI, HDI, GDP per capita, and the Democracy Index (overall score) were not correlated significantly with the BZSG.

Nevertheless we tested all models with and without log GDP per capita and HDI as predictors to verify if these variables significantly influence the findings.

### Multilevel Modeling

#### Baseline Model

As we expected, country characteristics significantly explain variation in BZSG at the individual level: an ICC of 0.25 means that the correlation between the BZSG scores of students within a country is 0.25. Mean national differences in BZSG scores (see Table 1) indicate that belief in a zero-sum game was strongest amongst people from Malaysia, Pakistan, Iran, India, Vietnam, and Nepal, and weakest amongst people from Portugal, Romania, Hungary, Brazil, and Colombia.

#### Random Coefficient With Individual-Level Predictors

As shown in Table 6, gender was positively correlated with BZSG, whereas age was negatively correlated. Youth and male gender were both positively associated with BZSG. According to the chi-squared difference test in Table 7, Model 2 provided a better fit to the data than Model 1.

**Table 6:** Multilevel models predicting of the BZSG.

	Model 1	Model 2	Model 3a	Model 3b	Model 4
Individual-level variables (L1)					
Gender (male)		0.09*	0.09*	0.09*	0.09*
Age		–0.02**	–0.02**	0.02**	–0.02**
Country-level variables (L2)					
Military expenditures			0.35*		0.20
Civil liberties				–0.72**	–0.56*
Random effects					
Residual	0.82	0.81	0.81	0.81	0.81
Gender	–	0.16	0.16	0.16	0.16
Intercept	0.27	0.29	0.26	0.27	0.26


*Number of observations = 5,520; Number of countries = 30. ^∗^*p* < 0.05. ^∗∗^*p* < 0.01.*

**Table 7:** Multilevel models fit indices.

Model	Type	Description	Δ df	–2 log likelihood	AIC	Δ χ^2^
1	Baseline (null) model	Individuals nested within their country with no other predictors	–	13,563.4	13,569.4	–
2	Random coefficient and fixed predictors	Individual (L1) level	4	13,509.1	13,523.1	54.3^∗∗^
3a	Random coefficient and fixed predictors	Individual (L1) and country (L2) level (militarization)	1	13,504.1	13,520.1	5.0^∗^
3b	Random coefficient and fixed predictors	Individual (L1) and country (L2) level (civil liberties)	1	13,501.5	13,517.5	7.4^∗∗^
4	Random coefficient and fixed predictors	Individual (L1) and country (L2) level (militarization and civil liberties)	2	13,499.9	13,517.9	4.8^∗^ (3a) 1.4 (3b)


*Number of observations = 5,520; Number of countries = 30. ^∗^*p* < 0.05. ^∗∗^*p* < 0.01.*

#### Random Coefficient With Country- and Individual-Level Predictors

Two individual-level (i.e., gender and age) and two country-level (i.e., military expenditure and civil liberties) predictors were tested sequentially in Models 3a and 3b. As reported in Table 7, both models offered a better fit than Model 2.

National military expenditure (Model 3a) and national level of civil liberties (Model 3b) emerged respectively as positive and negative predictors of BZSG at the individual level (see Table 6). However, results from Model 4 (which incorporated two fixed-effect predictors at the country level) showed that only civil liberties were independently—and negatively—associated with BZSG. As reported in Table 7, Model 4 was a better fit to the data than Model 3a, but not Model 3b^3^.

## Discussion

The aim of this paper was to investigate the relationships between BZSG, military expenditure, and civil liberties across different countries. The results support the hypotheses: military expenditure was positively correlated with individual BZSG, whereas electoral process and pluralism and civil liberties were negatively correlated.

Multilevel (MML) analyses showed that national levels of both military expenditure and civil liberties are associated with BZSG at the individual level. However, when including both variables in the model, only the national civil liberties score was associated (negatively) with individual BZSG, whereas the military expenditure was not significant. Therefore, our findings are in line with former studies indicating the importance of other factors in shaping militarization (such as the masculinity dimension: Hofstede et al., 2010).

In our models, we tested the relationship between country variables (military expenditure and civil liberties) and an individual variable (BZSG). Even though multilevel models include a line of causation from country to individual factors, with individual behavior being influenced by cultural context (e.g., the ecocultural framework, where socio-political context influences psychological variables; Berry, 1976), there is also the possibility that individual-and country-level factors are interdependent (Van de Vijver et al., 2008). The theory of social axioms (Bond et al., 2004) describes general social beliefs as reflections of a culture and the behavior of individuals socialized into that culture. That is why a multilevel approach was adopted. The links between socioeconomic systems and psychological processes seem to be very important in the context of what is currently happening around the world (e.g., Bar-On and Kassem, 2004).

These results suggest that the tested variables from both levels could be related to one another: when peaceful countries are attacked, their citizens start to believe that international relationships tend to be antagonistic, and so militarization increases. It is also possible that the influence is in the other direction: members of the government and others who influence budgetary decisions (e.g., increases in military expenditure) tend to believe that relationships are like a zero-sum game: public opinion and attitudes influence political institutions, which try to satisfy the needs of the society. The reverse is also true: although public attitudes are influenced by policymakers, military intervention can only be carried out when there is acceptance of war in the society (Foyle, 2004). It must be stated that military expenditure measures do not distinguish expenditures on international vs. civil conflicts, so the conclusions are limited to the general opinion (without differentiation on civil vs. international relationships).

In conclusion, the main findings reported in this manuscript are the correlations between aggregated scores for BZSG and indices of national military expenditure and democracy (i.e., civil liberties). Our analyses suggest the interdependence of individual-level beliefs and national-level objective macro indices, but without casual interpretations. We only suggest that if the majority of citizens saw the world as a source of common resources for everybody and endorsed a more harmonious vision of social relations, then perhaps relationships between countries would also be more balanced.

### Limitations and Future Research

A limitation of this study is that the sample was composed solely of university students, so there is a limit to how much can be said about the representativeness of the people from a country. However, this focus was similar across countries, so this helps somewhat with the ability to make comparisons across countries; also, these student data do provide an indication of the thinking of young elites.

Future research should seek to identify the beliefs which distort citizens’ perception of social relations, to identify some of the cultural variables influencing psychological outcomes, and to explain how variables on these two levels are related. It would also be worthwhile to conduct longitudinal studies, which would allow more causal interpretations. Our cross-sectional data do not allow for a clear statement that BZSG is responsible for militarization or is just a cultural adaptation to living in conflicted society. In addition, we had data from only 30 countries, where there were no external political conflicts. Therefore, our interpretations are limited to relatively stable and well-functioning societies, and student samples, living in a relatively affluent and urban environment, not experiencing severe threats.

At present, it is also unclear whether the effect operates at country level or at regional level. For example, the EU has made a concerted effort to organize military operations between nation states, thereby reducing militarization. While the model accounts for the nesting of participants in countries, it does not account for the fact that Greece’s militarization score is not independent from that of Belgium, as both are tied to the EU. In future studies, more data should be gathered according to regional and geopolitical criteria.

Another limitation is the BZSG scale used in the study, which comprises eight positively worded items. It is therefore likely that ratings from respondents from some nations would be more strongly affected by acquiescent responding than those from others, which could be a source of error in the conclusions drawn about national differences. We decided to use only eight items from the BZSG scale as we found some problems with negatively reworded items in a different sample in a previous study (see Różycka-Tran et al., 2015). However, the MGCFA analysis of equivalence was conducted, where measurement invariance of the BZSG scale was improved. In future studies, some positively worded items describing low belief in a zero-sum game could be created and incorporated into study.

It is further important and potentially beneficial to link individual-level data with national-level indices that have major importance for the wellbeing of the world. Our paper only presents correlations between the investigated relationships. Future study should establish causal relationships, which usually requires either longitudinal data or the use of priming studies.

## Author Contributions

JR-T and PJ conceived and designed the study, and contributed most of the text and references. JR-T was responsible for providing the overall idea and crafting the outline of the article. PJ assisted in crafting the thesis of the article and provided additional topic of discussion to implement within the text. MŻ-P and JP gathered BZSG scale data. JR-T and PJ delivered data concerning militarization, income, and democracy. PJ, MO, and JR-T prepared the data for analysis and conducted the data analysis. PJ and MO performed the statistical and multilevel analysis. JR-T, PJ, MO, and MŻ-P wrote the manuscript. PJ visualized the data. All authors were involved in the revisions of the manuscript.

## Conflict of Interest Statement

The authors declare that the research was conducted in the absence of any commercial or financial relationships that could be construed as a potential conflict of interest.

## Footnotes

1 The students filled out the BZSG scale as part of a broader research project including other measures of subjective well-being: Personal Well-being Index, Satisfaction with Life Scale, Positive and Negative Affect Schedule, and two scales measuring Entitlement Attitudes (see Różycka-Tran et al., 2018b; Żemojtel-Piotrowska et al., 2018). We tried to include countries from all continents, differentiated in culture, economics and politics. However, we selected samples comparable in gender, sex and age, where measurement invariance of BZSG was confirmed, also where GDP, HDI or democracy index was available.

2 In current study only 8-item positively worded BZSG scale was used. However, the issue of acquiescent responding was investigated in earlier studies using the 12-item BZSG scale, including four reverse-worded items (see Różycka-Tran et al., 2015). To exclude cultural response style (that the negatively worded items could be biased culturally by acquiescence bias; see Smith, 2004), authors noted that the originally used in their studies 12-item BZSG scale was not significantly correlated with the acquiescence index constructed by Smith (*r* = 0.107, *p* = 0.64, *n* = 21) nor were the 8-item (*r* = 0.27, *p* = 0.23) or 4-negatively worded items (*r* = –0.27, *p* = 0.23) scales.

3 log GDP per capita (-0.04, *p* = 0.90) and HDI (0.07, *p* = 0.96) of the country were not significant predictors of the BZSG, taking into account age (-0.02, *p* < 0.01) and gender (0.09, *p* < 0.05) at L1; and civil liberties (-0.54, *p* < 0.05) with militarization (0.19, *p* = 0.25) at L2 (-2 log likelihood = 13,499.9; AIC = 13,521.9). So, we decided to report results from analyses without these control variables to avoid biased finding (see Becker et al., 2016).
